# Complications Following Expander/Implant Breast Reconstruction Utilizing Acellular Dermal Matrix: A Systematic Review and Meta-Analysis

**Published:** 2011-11-03

**Authors:** Ian C. Hoppe, Janet H. Yueh, Cindy H. Wei, Naveen K. Ahuja, Priti P. Patel, Ramazi O. Datiashvili

**Affiliations:** Department of Surgery, Division of Plastic Surgery, New Jersey Medical School—University of Medicine and Dentistry of New Jersey

## Abstract

**Background:** The recent increase in popularity of acellular dermal matrix assistance in immediate expander/implant breast reconstruction has led to variety of viewpoints. Many studies are published indicating an increase in complications with the use of acellular dermal matrix, while others indicate there is no increase in complications. **Methods:** This meta-analysis utilizes information from available studies that directly compare one specific type of acellular dermal matrix with traditional methods of immediate expander/implant breast reconstruction. Eight studies were found through a meticulous literature search that met these criteria. **Results:** There was more than a 2-fold increase in the number of infections and explanations in the acellular dermal matrix group compared to the control. There was a 3-fold increase in seroma formation in the acellular dermal matrix group compared to the control. There was a significant difference of intraoperative fill volumes between the acellular dermal matrix group compared to the control. **Conclusions:** This study illustrates that after pooling all available date regarding the use of acellular dermal matrix in immediate expander/implant breast reconstruction there appears to be an increased rate of complications. However, the increased intraoperative fill volume may lead to ultimately greater patient satisfaction.

The use of AlloDerm (LifeCell, Branchburg, New Jersey), an acellular dermal matrix (ADM), in breast reconstruction to facilitate complete coverage of the implant/expander gained popularity in 2005.^1^ The introduction of this biomaterial has made it possible to provide complete tissue expander coverage without dissection of the serratus anterior or rectus abdominis muscle/fascia. In addition, it has been shown that when using ADM, the intraoperative tissue expander fill volume was increased and the total number of expansions needed was decreased.^2^ However, ADM use does not come without complications; increased rates of infection and seroma formation have been linked with ADM use.^3^^-^5

Five years following the adoption of ADM in expander/implant breast reconstruction, it is still unclear whether its use presents an increased risk of infection and/or seroma formation. The results of most individual studies are oftentimes difficult to interpret. The authors believe that a more definitive conclusion may be drawn by examining these studies through a systematic review and pooling data from these studies into a meta-analysis. Selected studies were examined for rates of complications in patients undergoing ADM-assisted and conventional expander/implant breast reconstruction. A literature search was performed to select high-quality observational studies, the highest level of evidence currently available, that examine the relevant data. The level of evidence of the resulting systematic review of these studies is classified as 3a (Based on the University of Oxford's Centre for Evidence Based Medicine levels of evidence^6^). Systematic reviews are usually based on randomized controlled trials to achieve a level of evidence of 1a. Because of the nature of the studies in this systematic review, Stroup et al's consensus article regarding meta-analyses of observational studies was used in the reporting of methods and results of this study.^7^ This method has been validated previously for the reporting of observational studies in the field of plastic and reconstructive surgery.^8^ Guidelines compiled specifically for systematic reviews and meta-analyses in plastic surgery literature were also followed.^9^

## Search Strategy

**Figure 1:**
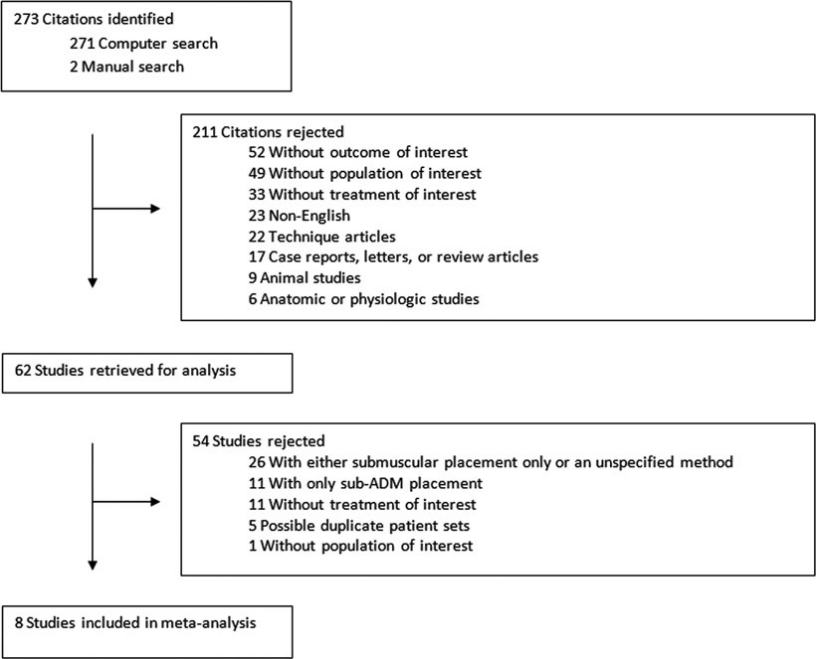
Study acquisition.

One author (I.C.H.) conducted all initial searches. PubMed was searched with the keywords, “alloderm,” “biocompatible materials,” “acellular dermal matrix,” “breast,” “expander implant,” and “infection” through February 2011. Daily updates of new papers that matched the search criteria were provided by e-mail. The Cochrane Central Register of Controlled Trials revealed 4 ongoing studies examining the use of ADM in breast reconstruction. In addition, reference lists were scrutinized to find any studies that may have been inadvertently excluded in the initial search. Abstracts were initially used to select relevant articles utilizing inclusion criteria (Table 1). Full-text articles were retrieved and submitted to the exclusion criteria (Table 2). A diagram of the search process is presented in Figure 1. In addition a brief literature search was performed to identify several other ADM products and their clinical applications.

## DATA EXTRACTION

All studies included in the meta-analysis evaluate the rates of complications in ADM-assisted compared to traditional implant/expander breast reconstruction following mastectomy. All data were extracted directly from each study. Two researchers evaluated and independently extracted data from each study using a standardized form. The researchers were not blinded to the study being examined as this has been shown to be unnecessary.^10^

## STATISTICAL ANALYSIS

Statistical analysis was performed utilizing Review Manager (RevMan [Computer program], Version 5.0, Copenhagen: The Nordic Cochrane Centre, The Cochrane Collaboration, 2008). A fixed effect model and the Mantel-Haenszel test was utilized to provide pooled odds ratios (ORs) for the variables under examination.

## RESULTS

Seven observational studies were found to fit the inclusion and exclusion criteria.^2^^-^4^,^^11^^-^14 One study^5^ reported only explantation rates and was only included in that analysis. Table 3 provides a summary of the characteristics of each study.

In general, 3 studies^2^^,^^11^^,^^12^ reported no difference in the rate of complications and 4 studies^3^^,^^4^^,^^13^^,^^14^ reported an increased rate of complications between groups.

## INCLUDED STUDIES

### Antony et al^13^

This retrospective comparative study examines immediate 2-stage tissue expander breast reconstruction over a 4-year period at a single institution. In this period, there were 153 breasts included in the ADM (AlloDerm) group and 2910 breasts included in the control group. The control group was defined as a traditional musculofascial method. Descriptive characteristics are provided in Table 4. Notably, the mean BMI (body mass index) for the non-ADM group was lower than the ADM group and the rate of preoperative radiation therapy was higher in the ADM group. No formal comparison between ADM and control groups was performed. Outcomes examined included seroma, cellulitis, hematoma, and premature explantation of the expander. Overall, there was an increased incidence of complications noted in the ADM group. Age, BMI, and axillary dissection were determined to be independent risk factors for development of one or more complications.

### Chun et al^3^

This retrospective comparative study examines immediate breast reconstruction utilizing tissue expanders and implants over a 6-year period at a single institution. During this period, there were 269 breasts included in the ADM (AlloDerm) group and 146 breasts in the control group. The control group was defined as expanders/implants with total submuscular coverage or partial submuscular coverage with corresponding partial subcutaneous coverage. Within this group, 68 latissimus dorsi and 1 pedicled transverse rectus abdominis muscle flaps were included because they utilized a tissue expander or an implant. Descriptive characteristics are provided in Table 5. All characteristics were compared to find significant differences among groups. Notably, there was a significant (*P* = .002) difference between the BMI of the 2 groups and the mastectomy specimen (*P* < .001) weight of each group. Outcomes examined included hematoma, seroma, necrosis, intraoperative fill volume, and infection. Infection was broken down into minor (successfully treated with outpatient antibiotics) and major infections (required admission), both of which were combined to determine the rate of infection. Overall, there was an increased rate of complications in the ADM group. Body mass index and the use of ADM were determined to be significant risk factors for the development of infection and seroma.

### Lanier et al^4^

This retrospective comparative study examines immediate 2-stage tissue expander breast reconstruction over a 3-year period at a single institution. During this period, 52 breasts were included in the ADM (AlloDerm) group and 75 breasts in the control group. The control group was defined as the creation of a subpectoral pocket and a lateral pocket utilizing serratus anterior muscle. Descriptive characteristics are provided in Table 6. All characteristics were compared to find significant differences among groups. Significant differences were noted in BMI (*P* < .001) and mean breast tissue removed (*P* = .005) between groups. Outcomes examined included seroma, hematoma, necrosis requiring revision, capsular contracture, intraoperative fill volume, and cellulitis or infection. Overall, there was an increased rate of complications in the ADM group.

### Liu et al^14^

This retrospective comparative study examines immediate 2-stage tissue expander breast reconstruction over a 5-year period at a single institution. During this period, 266 breasts were included in the ADM (AlloDerm) group and 204 breasts in the control group. The control group was defined as either total or partial submuscular placement of the expander. Descriptive characteristics are provided in Table 7. Significant differences were noted in mean breast tissue removed (*P* = .0184). Outcomes included infection, implant removal, skin flap necrosis, seromas, intraoperative fill volume, and hematomas. Overall, complications were higher in the ADM group.

### Nahabedian^11^

This retrospective comparative study examines breast reconstruction utilizing prosthetic devices over a period of 11 years. Specifically, this study examined the rates of complications with and without chemotherapy and/or radiation. During this period, 100 breasts were included in the ADM (AlloDerm) group and 376 breasts were included in the control group. The control group was defined as device placement beneath the pectoralis major and lower mastectomy skin flap. Few descriptive characteristics were included regarding the patients. Outcomes examined included infection, implant removal, and ADM removal. Overall, there was no difference in the rate of complications between groups.

### Preminger et al^12^

This matched, retrospective cohort study examines immediate tissue expander implant breast reconstruction over a 2-year period. Matching criteria included median expander size, history of radiation, and indication for mastectomy. Matched cohorts of 45 breasts each were prepared. The experimental group utilized ADM (AlloDerm). The control group was defined as creation of a subpectoral pocket with elevation of the serratus anterior and superior rectus abdominis muscle/fascia. Few descriptive characteristics were included regarding the patients, but due to the matched cohort nature of the study, it can be assumed that there are likely few differences between groups. Outcomes examined included seroma, hematoma, intraoperative fill volume, and cellulitis. For the purposes of the meta-analysis, the rate of cellulitis was used as the rate of infection. Overall, there was no difference in the rate of complications between groups.

### Sbitany et al^2^

This retrospective comparative study examines tissue expander implant breast reconstruction over a 4-year period. During this period, 92 breasts were included in the ADM (AlloDerm) group and 84 breasts were included in the control group. The *control* was defined as placement in a subpectoral pocket with elevation of the serratus anterior laterally. Descriptive characteristics are provided in Table 8. No significant differences were found between these characteristics. Outcomes examined included seroma, cellulitis, intraoperative fill volume, and infection requiring expander removal. For the purpose of this meta-analysis, the rate of cellulitis was used as the rate of infection. Overall, there was no difference in the rate of complications between groups. During the reporting of complications, the authors used the number of patients instead of the number of reconstructions. The decision was made to utilize these values in terms of the number of reconstructions for the purpose of this meta-analysis to avoid discrepancies in reporting.

## META-ANALYSIS OF STUDIES

**Figure 2:**
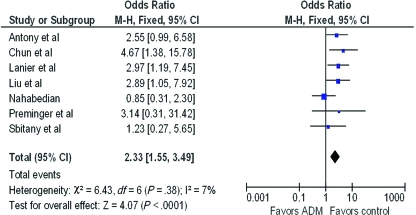
Forest plot of rate of infections between groups. ADM indicates acellular dermal matrix.

**Figure 3:**
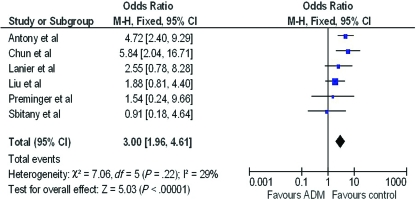
Forest plot of rate of seromas between groups. ADM indicates acellular dermal matrix.

**Figure 4:**
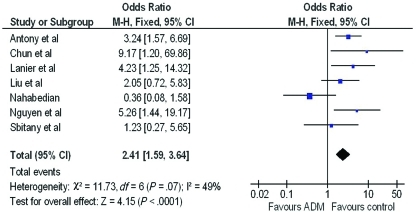
Forest plot of rate of explantations between groups. ADM indicates acellular dermal matrix.

**Figure 5:**
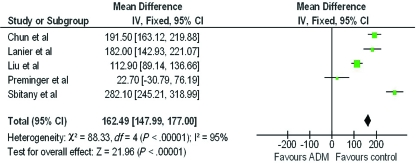
Forest plot of mean intraoperative tissue expander fill volume. Mean standard deviation from the other studies were used if no standard deviation was provided. ADM indicates acellular dermal matrix.

A forest plot of the OR of infection across all studies is provided in Figure 2. The meta-analysis reports over a 2-fold increase in rate of infections for the ADM-assisted group (OR of 2.33; 95% confidence interval [CI], 1.55-3.49). A forest plot of the OR of seroma formation across the 5 studies reporting this statistic is provided in Figure 3. There was a 3-fold increase in the incidence of seromas in the ADM-assisted group (OR of 3.00; 95% CI, 1.96-4.61). A forest plot of the OR of tissue expander explantation across all studies is provided in Figure 4. The meta-analysis reports over a two-fold increase in rate of explantations for the ADM-assisted group (OR of 2.41; 95% CI, 1.59-3.64). A forest plot of the mean difference of intraoperative fill volumes of the tissue expanders is provided in Figure 5. The meta-analysis reports a mean difference of 162 mL (95% CI, 148-177)

## DISCUSSION

As breast reconstruction following mastectomy utilizing ADM becomes an acceptable reconstructive option, it is important to understand the risks and benefits of its use. In the case of expander/implant breast reconstruction, several variables are important to the end result and ultimately patient satisfaction. These include postoperative complications such as infections, seromas, and the rate of expander explantation following one of these complications. It has been previously found that the use of ADM in postmastectomy immediate reconstructions results in high levels of patient satisfaction.^15^

While there is much in the current literature regarding ADM use in expander/implant breast reconstruction, the majority focuses on the rates of complications and the dynamics of expansion.^1^^,^^16^^-^21 Unfortunately, most of these publications are case series with no control group.

It is oftentimes difficult to evaluate the results of each individual study due to the presence of confounding factors such as BMI, surgeon skill (both plastic surgeon and breast surgeon), and unclear methods of study design. The goal of a systematic review and meta-analysis is to utilize samples from different studies to minimize these confounding variables. The 7 studies included in this analysis were split regarding whether complication rates were higher in the ADM group. This presents a problem for the surgeon attempting to determine the true risks and benefits of the use of ADM. It is the goal of this meta-analysis to pool all available data to obtain a clearer picture of the risks inherent with the use of ADM in expander/implant breast reconstruction.

This meta-analysis demonstrates that ADM use in expander/implant reconstruction results in increased rates of infection, seroma, hematoma, and explantation compared to a control. This is not conceptually difficult to understand given that ADM is a foreign body, despite its biologic properties. Foreign bodies incite an inflammatory reaction that results in increased rates of infection. Furthermore, Alloderm is considered an aseptic product as opposed to sterile, providing a further risk of postoperative infection. This may explain why its use is associated with an increased risk of infection despite the simultaneous use of tissue expanders and implants, also foreign bodies. The difference is that the tissue expanders and implants are sterile prior to introduction. The advantage provided by ADM is the eventual ingrowth of vasculature that has the potential to provide the means for eliminating bacterial infection,^11^ although this process takes time leaving a period of time where the ADM may be susceptible to infection. This does not preclude the use of ADM in breast reconstruction as the benefits may outweigh the risks reported by this study. These benefits may include decreased postoperative pain and morbidity, decreased operative time, increased initial expander fill volume, and an increased rate of expansion. This meta-analysis showed a significant difference between the ADM group and the control regarding intraoperative fill volume. This may lead to a fewer number of expansions and a earlier expander/implant exchange procedure as evidenced in one of the studies.^2^

Weaknesses of this meta-analysis include the possible introduction of publication bias and a slightly different definition of outcome measurements across studies. Through our meticulous literature search we attempted to include all published data on the topic. Unfortunately, researchers are less likely to publish unfavorable results, introducing a degree of bias. An attempt was made to generalize outcome measurements throughout each study as described in each synopsis. For instance, not all studies provided a strict definition of *seroma*. This is a potential source of bias due to the fact that there are 2 types of seromas, those which do not require treatment and those which do. While this study only examined one brand of ADM, many other types exist. Products differ in dermal origin (human/porcine), donor screening process, and method of preparation. A brief description of commonly used dermal matrices used in breast reconstruction and abdominal wall repair is illustrated in Table 9.^22^^-^27 Further studies will need to be performed to compare the efficacy of these products in breast reconstruction. As of now it is unclear how the different processing methods and dermal origins may impact the complication rate in immediate tissue expander implant breast reconstruction.

### Clinical applications

While the finding that there is an increased rate of complications when ADM is utilized does not preclude its use in breast reconstruction, it brings up several important points. It is important for the surgeon to be knowledgeable regarding the rates of possible complications so that this information may be passed on to patients in a way that allows them to make the most educated decision. On the same token, it is imperative to explain the strengths inherent with the use of ADM in breast reconstruction. Less time is needed to spend raising muscle flaps and the patient may subsequently experience less postoperative pain when utilizing ADM.

As in any reconstructive procedure, the surgeon must use their judgment when deciding on an operative plan. This study simply attempts to clear up an intensely debated issue in breast reconstruction. For a more definitive conclusion to be reached, a large multicenter, randomized controlled trial must be organized examining not only the complications following ADM use, but the benefits as well.

## Figures and Tables

**Table 1:** Inclusion criteria

Clinical human study
Postmastectomy
Breast reconstruction
AlloDerm utilized
English language

**Table 2:** Exclusion criteria

Method other than tissue expander/implant breast reconstruction
No comparison between ADM group and control
Previously published data

**Table 3:** Characteristics of each study included

Source		No. of breasts	Mean age, y	Mean body mass index	Infections, n (%)	Seromas, n (%)	Hematomas, n (%)	Explantation, n (%)
Antony et al	ADM	153	44.5*	23.8	5 (3.3)	11 (7.2)	3 (2.0)	9 (5.8)
	Control	2910	48.1*	26.3	38 (1.3)	47 (1.6)	26 (0.9)	55 (1.9)
Chun et al	ADM	269	47	25.5	24 (8.9)	38 (14.1)	6 (2.2)	16 (5.9)
	Control	146	46.2	23.8	3 (2.1)	4 (2.7)	2 (1.4)	1 (0.7)
Lanier et al	ADM	52	51	29.8	15 (28.9)	8 (15.4)	0 (0)	10 (19.2)
	Control	75	50	24.7	9 (12)	5 (6.7)	0 (0)	4 (5.3)
Liu et al	ADM	266	NA	24.9	18 (6.7)	19 (7.1)	1 (0.4)	13 (4.8)
	Control	204	NA	24.8	5 (2.4)	8 (3.9)	0 (0)	5 (204)
Nahabedian	ADM	100	46	NA	5 (5)	NA	NA	2 (2.0)
	Control	376	NA	NA	22 (5.85)	NA	NA	20 (5.3)
Preminger et al	ADM	45	NA	NA	3 (6.7)	3 (6.7)	1 (2.2)	NA
	Control	45	NA	NA	1 (2.2)	2 (4.4)	0 (0)	NA
Sbitany et al	ADM	92	48.6	26.4	4 (8)	3 (6)	NA	4 (4.3)
	Control	84	51.7	28.2	3 (6)	3 (6)	NA	3 (3.6)	

ADM indicates acellular dermal matrix; BMI, body mass index; NA, not available.

*Median.

**Table 4:** Characteristics of Antony et al

	ADM	Control
Median age	44.5	48.1
Mean BMI	23.8	26.3
Adjuvant/neoadjuvant chemotherapy, %	45.8	50.1
Preoperative radiation, %	15.6	11.1
Postoperative radiation, %	9.4	11.4

ADM indicates acellular dermal matrix; BMI, body mass index.

**Table 5:** Characteristics of Chun et al

	ADM	Control
Mean age	47	46.2
Mean BMI	25.5	23.8
Preoperative chemotherapy, %	14.9	8.2
Postoperative chemotherapy, %	19	30.8
Preoperative radiation, %	8.7	5.2
Postoperative radiation, %	8.6	6.5
Mean mastectomy specimen weight, g	577.2	389.9

ADM indicates acellular dermal matrix; BMI, body mass index.

**Table 6:** Characteristics of Lanier et al

	ADM	Control
Mean age	51	50
Mean BMI	29.8	24.7
Preoperative chemotherapy, %	11.5	20
Postoperative chemotherapy, %	51.9	45.3
Preoperative radiation, %	5.8	9.3
Postoperative radiation, %	5.8	10.7
Mean mastectomy specimen weight, g	646	984

ADM indicates acellular dermal matrix; BMI, body mass index.

**Table 7:** Characteristics of Liu et al

	ADM	Control
Mean BMI	24.9	24.8
Radiation, %	9.8	10.4
Mean mastectomy specimen weight, g	526.4	456.9

ADM indicates acellular dermal matrix; BMI, body mass index.

**Table 8:** Characteristics of Sbitany et al

	ADM	Control
Mean age	48.6	51.7
Mean BMI	26.4	28.2
Postoperative radiation, %	12	6

ADM indicates acellular dermal matrix; BMI, body mass index.

**Table 9:** Commonly used dermal matrices

Brand Name	Manufacturer	Donor	Donor Screening	Hydration Status	Description	Used in Expander/Implant Breast? Reconstruction?
Alloderm	LifeCell	Human	Own screening modality	Dehydrated	Processed to remove epidermis and dermal cells	Yes
DermaMatrix	Synthes	Human	MTF screening	Dehydrated	Processed to remove epidermis and dermal cells	Yes
FlexHD	Ethicon	Human	MTF screening	Hydrated	Processed to remove epidermis and dermal cells	Yes
Permacol	Covidien	Porcine	n/a	Hydrated	Processed to remove cells; resulting collagen cross-linked	No
Strattice	LifeCell	Porcine	n/a	Dehydrated	Processed to remove cells	Yes

MTF indicates Musculoskeletal Transplant Foundation.

*According to official product description.
